# Unexpected Gastrointestinal Tract injury years following Laparoscopic Adjustable Gastric Banding

**DOI:** 10.1016/j.ijscr.2020.11.023

**Published:** 2020-11-07

**Authors:** Subhi Mansour, Giuseppe Borzellino, Yoram Kluger, Safi Khuri

**Affiliations:** a General Surgery Department, Rambam Health Care Campus, Haifa, Israel; b HPB and Surgical Oncology Unit, Rambam Health Care Campus, Haifa, Israel; c General Surgery Department, University of Hospital Verona, Verona, Italy

**Keywords:** LAGB, Laparoscopic Adjustable Gastric Band, CT, Computed tomography, Case report, Laparoscopic Adjustable Gastric Band, Bariatric surgery, Severe pressure effect, Life threatening complications

## Abstract

**Introduction:**

Band migration is a late complication of Laparoscopic Adjustable Gastric Banding insertion, although rare it could be life threatening presenting as peritonitis secondary to gastro-intestinal tract injuries. A case of an unexpected extension of severe gastro-intestinal tract injuries secondary to intra-gastric migration and distal band dislocation is reported.

**Presentation of case:**

A 53 years old male, with a history of laparoscopic gastric banding 15 years before and known erosion of the band into the gastric lumen was admitted for abdominal pain and raised serum amylase. Imaging revealed dislocation of the band down to the jejunum.

Endoscopy and exploratory surgery showed severe decubitus pressure on the gastric antrum up to the duodenum as well as on the pancreas due to rod-like effect of the gastric band catheter and multiple sites of perforation on distal duodenum and small bowel proximal to the band, which migrated within the lumen until 90 cm distal to the Treitz ligament. Extended distal gastrectomy and resection of distal duodenum and small bowel extended to the proximal affected small bowel were necessary. Digestive tract was restored by a gastro-jejunostomy and duodeno-jejunostomy in a Roux-En-Y configuration with duodenal stump closure on tube duodenostomy. A post-operative leakage from the duodenal stump was treated conservatively and the patient was discharged on post-operative day 21.

**Discussion:**

Erosion and migration of the band within the digestive lumen is one of the less frequent late complications occurring after LAGB, furthermore, the amount of extensive damage reported in this case presentation has yet to be reported in literature.

**Conclusion:**

Migration of the band should be considered in the differential diagnosis of abdominal complain in patients with adjustable gastric banding. Such a complication could be severe, and lesions may have unexpected extension requiring complex surgical approach.

## Highlights

• Although most complications following LAGB are confined to the stomach and port site, some may envolve the entire bowel causing extensive damage.
• One must be wary of the presentation of long term complications, as it may initially present as an unrelated pathology, such as acute pancreatitis.
• Long term complications may be asymptomatic for years despite extensice damage, including multiple contained perforations.
• The surgical procedure performed to treat long term complications should be planned and performed by physicians experienced in foregut surgery.

## 1 Introduction

Laparoscopic Adjustable Gastric Banding (LAGB), firstly described by Belachew in 1993 [1], is a purely restrictive bariatric surgery. It is the simplest form of the minimally invasive surgical procedures done for obesity, but has also become less practiced due to high rate of revision secondary to late weight gain and complications [2]. Early results for weight loss were promising, but long-term results are less encouraging [3], [4], [5], [6], not only due to the reported failure rate of long-term weight loss, but also late complications, both requiring in up to 67% of cases re-intervention at a 14 years follow up [7]. Late complications following LAGB are more frequent than early complications and they include pouch dilation, band slippage, band erosion and device-related complications [2], [8]. Life threatening major complications presenting as massive gastrointestinal haemorrhage, obstruction or perforation are rare and require prompt surgical procedures [2].

Herein, we present a unique case of gastro-intestinal tract injury along the path of an unexpected extension of the band secondary to erosion and distal intraluminal migration of a gastric band 15 years following its positioning for bariatric surgery purpose.

## 2 Case report

A 53-years-old white male patient was admitted to our Emergency Medicine department with complaints of left abdominal pain of one-day duration, with worsening on the day of admission. Nausea, vomiting and chills accompanied the pain. His past medical history includes hypertension and type 2 obesity for which, LAGB operation was performed 15 years prior. He was known to have non-functioning gastric banding for 8 years due to intra-gastric erosion of the band with failed trial of upper endoscopic removal. In addition, the patient had undergone laparoscopic cholecystectomy 5 months prior to admission due to symptomatic cholelithiasis (episode of mild biliary pancreatitis treated conservatively). The patient is a non-smoker with no known allergies.

On physical examination upon his admission, his vital signs were within normal limits. His BMI was 37.7. Abdominal examination demonstrated a soft abdomen, with left upper abdominal slight tenderness. Digital rectal exam was normal. No signs of port infection were observed. Complete blood count showed increased white blood cells of 21,000, of which 80% were neutrophils with 7% bands. His liver and kidney function tests were within normal limits. Serum and urinary Amylase levels were highly elevated at more than 3 folds the normal range (serum amylase 336 U/L, urinary amylase 3281 U/L). Due to these findings, the patient was admitted with clinical and laboratory diagnosis of Acute Pancreatitis, for conservative management.

**Fig. 1:**
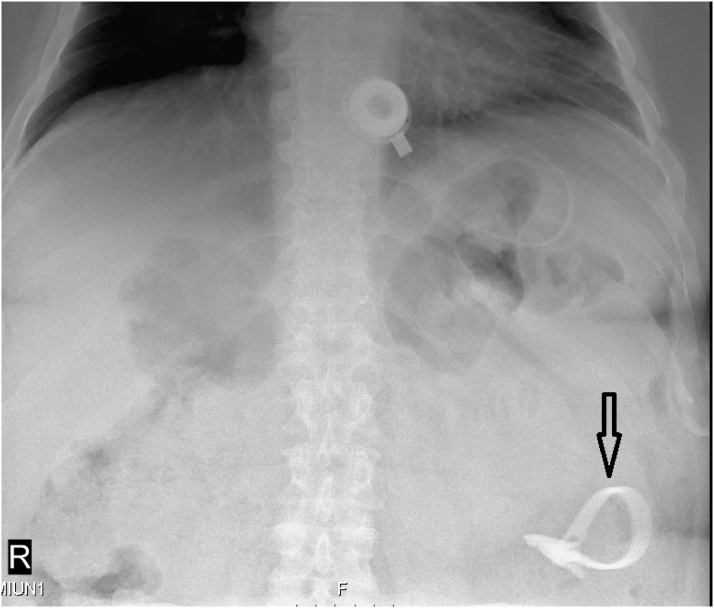
Plain abdominal X-ray showing gastric band localized to the left lower quadrant (arrow).

**Fig. 2:**
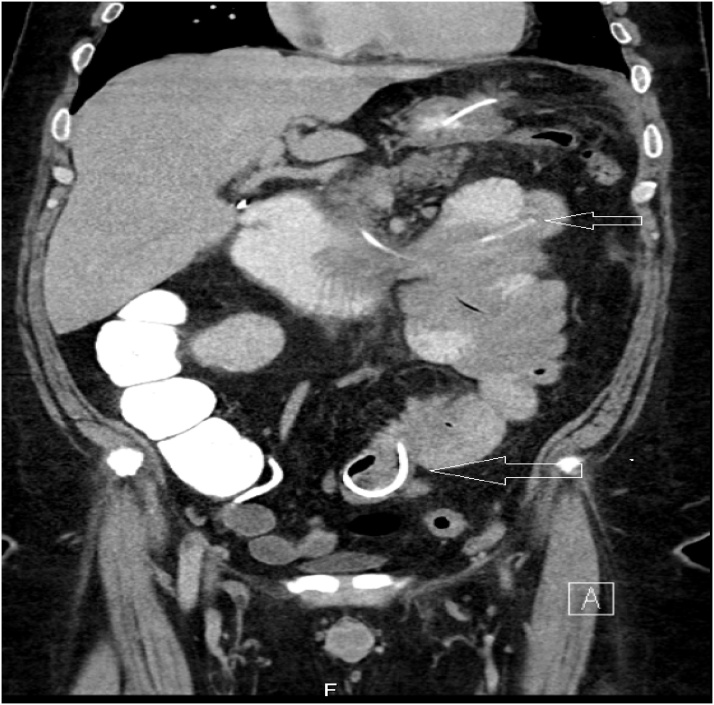
On Coronal abdomino-pelvic CT scan, the gastric band is localized in the upper jejunum (long arrow), with the catheter (short arrow) passing through the upper gastrointestinal tract. Thickened small bowel wall, as well as surrounding fat haziness is also seen.

13 hours following admission, the patient complained of abdominal pain exacerbation despite adequate pain control regime, along with a documented fever of 38.8 c. Repeated abdominal exam revealed localized left mid abdominal peritonitis, without palpable masses. Revision of a plain abdominal X-ray done several months prior (former admittance due to biliary pancreatitis) demonstrated gastric band localized in the left lower quadrant of the abdomen, and therefore, distal migration was suspected (Fig. 1). A Computed Tomography (CT) scan was obtained, which showed a port located subcutaneously at the upper abdominal wall, attached to the catheter which enters the stomach and passes through the duodenum distally to the upper part of the jejunum, where the gastric band was located (Fig. 2). The wall of the small bowel surrounding the catheter and the band was thickened with haziness of the nearby fatty tissues. No free intra-abdominal air or fluid was demonstrated.

**Fig. 3:**
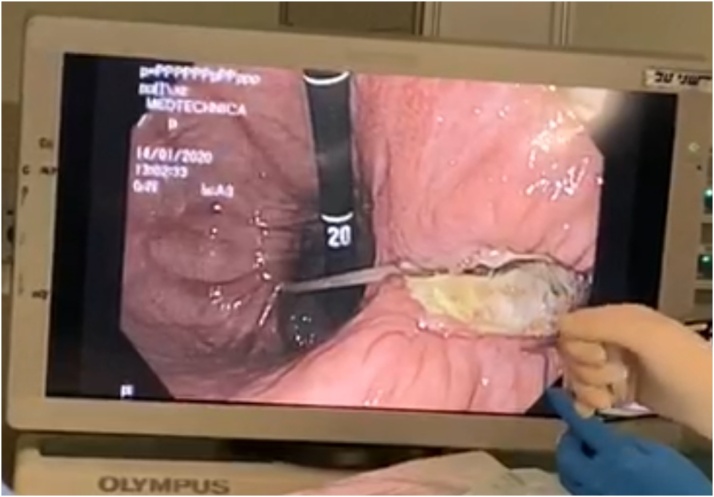
Intra-operative upper Endoscopy demonstrating severe decubitus pressure changes involving the gastric antrum and duodenum due to rod like effect of the gastric band catheter.

**Fig. 4:**
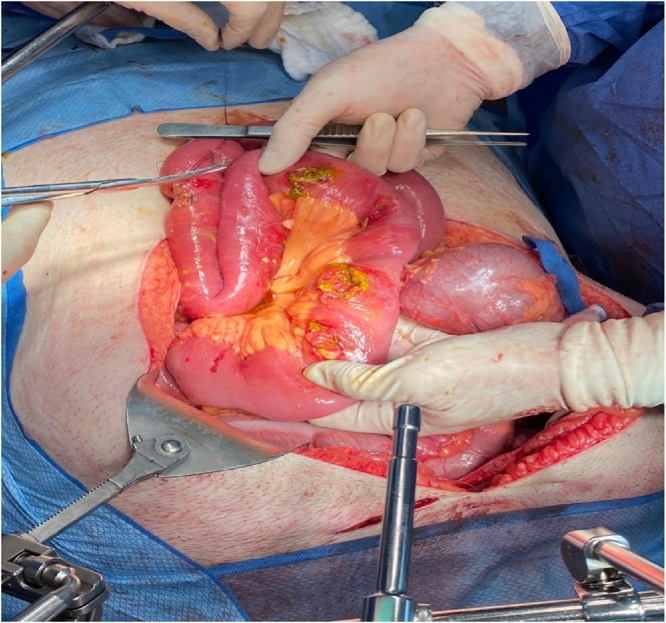
On exploration of the abdomen, multiple perforation sites of different diameter were demonstrated along the upper jejunum.

**Fig. 5:**
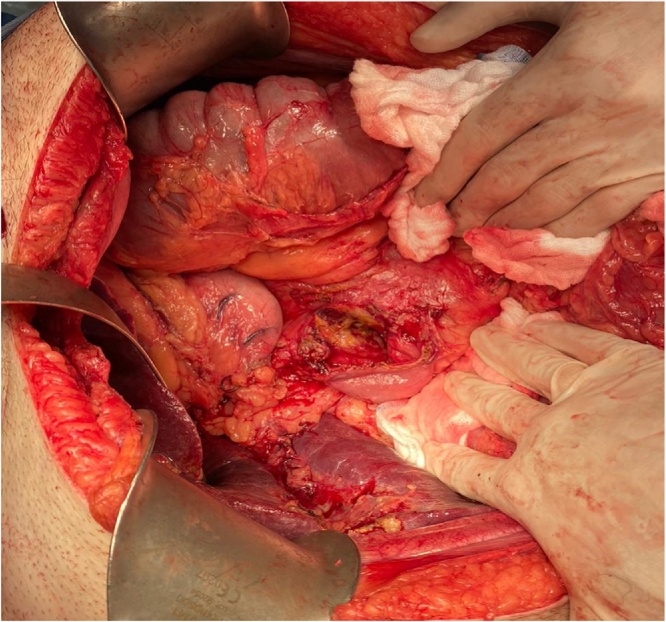
Figure 5 demonstrates severe pressure effect involving the posterior and medial wall of the duodenum, following distal gastrectomy.

**Fig. 6:**
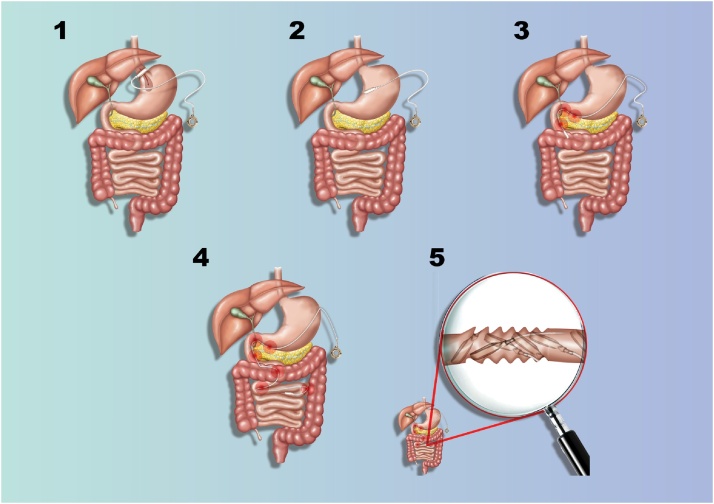
This figure demonstrates the hypothesized journey of the gastric banding in the upper gastrointestinal tract.

Due to these findings, the patient underwent diagnostic laparoscopy, performed by the head of the department, with intra-operative upper endoscopy. On intra-operative upper endoscopy, necrosis of the medial wall of the gastric antrum, through to the second part of the duodenum due to chronic severe decubitus pressure changes was demonstrated. These changes were the result of a rod-like effect of the band catheter, being fixed at the proximal (port) and distal (band) sides (Fig. 3). A subsequent diagnostic laparoscopy demonstrates small amount of turbulent-serous fluid at the left upper abdomen, with a zigzag configuration of the first 90 cm of the jejunum. On alignment of the jejunum, multiple opposing perforation sites of similar diameters (7 mm) on the lateral wall were observed, most probably due to pressure effect. This was followed by an exploratory laparotomy, during which the catheter was cut, an enterotomy was performed in order to remove the band, followed by the removal of the port with the cut catheter. Upon entering the lesser sac following gastro-colic ligament dissection, a severe chronic inflammation involving the antrum and the postero-medial duodenal wall was noticed due to posterior perforation of the stomach. The same findings were also found involving the head of the pancreas due to the previously mentioned rod-like effect of the gastric band. In addition, 9 perforation sites along the first 90 cm of the jejunum were noticed (Fig. 4). Due to the aforementioned findings, resection of the upper jejunum was completed along with a distal gastrectomy. Following resection of the small bowel and mobilization of the 3rd and 4th duodenal parts, two additional perforation sites were noticed, thus resection of these duodenal parts were done. Reconstruction of the GI tract was completed by gastro-enterostomy and duodeno-enterostomy with a Roux-En-Y configuration. The proximal duodenal stump closed primarily following the insertion of duodenostomy tube due to severe pressure effect involving the posterior and medial wall (Fig. 5). The papilla of Vater was uninvolved. The hypnotised theory for the aforementioned injury is internal penetration and distal migration of the gastric band causing rod-like pressure necrosis of the involved organs (Fig. 6).

Post-operative period was complicated by a duodenal stump leak, treated successfully by conservative means including Nothing Per Os (NPO), Total parenteral nutrition (TPN), intravenous somatostatin analogues and antibiotics. The patient was discharged home on post-operative day 21. On 3 weeks follow up at the outpatient clinic, the patient was doing well without any complaints and the duodenostomy was removed.

The pathology report showed stomach wall with ulceration and peritonitis and small bowel with foci of ulceration and necrosis of intestinal wall, ulcers were similar in size and shape suggesting mechanical cause.

## 3 Discussion

Erosion and migration of the band within the digestive lumen is one of the less frequent late complications occurring after LAGB, [9]. Moreover, migration is generally limited to the stomach and cases with distal dislocation of the band have been only a matter of case reports [10], [11], [12], [13], [14] or description of cases within case series [15], [16]. Life threatening presentations of such distal migration of the band causing gastro-intestinal injuries are very rare [15], [16], [17]. A case of erosion with extensive gastro-intestinal injury secondary to distal dislocation of the band, 15 years after LAGB for obesity and 8 years after a diagnosis of an intra-gastric migration is hereby reported. The case is of interest since the initial presentation oriented toward an acute pancreatitis. Secondly, to the best of our knowledge, this is the first case that reports such extensive gastro-intestinal tract injuries with skip lesions from the antrum to the distal Jejunum at 90 cm distal to the Treitz ligament.

The rate of band erosion incidence has been reported to have a median value of 3.9% with extreme value of 0.8%–28% on a review based on 17 selected studies [9]. The wide range of values may be explained by the heterogeneity of selected studies, all published before 2014, with different follow up duration. For example, different studies included patients operated on using two different techniques, the peri-gastric and the pars flaccida technique. Furthermore, two different first and second generation bands were used in different cases. Band erosion has been reported to be more frequent with the peri-gastric technique compared to the pars flaccida technique at 1% vs 0.4% (p < 0.001) respectively in a retrospective comparative study [18]. Additionally, first generation band are more likely to be associated with band migration compared to second generation bands as reported in an another comparative but non randomized study [19] with respective rate of 0.37% versus 0.04% (p = 0.0084). More recent case series [6], [16], [19], [20], [21] have reported a rate of band erosion between 0.04% and 4.6% according to various follow up periods and different surgical techniques [6], [16].

According to the duration of follow up, a systematic review on erosion after LAGB [22] reported rates of erosion ranging from 0.23% up to 32.65% with maximum follow up of 9 years. In a more recent systematic review [9] follow up were reported for up to 18 years but no timing relation with erosion occurrence was reported. A more recent case report described erosion 18 years after LAGB [23]. Another 2 case reports described the complications late after LAGB at 13 years [13] and at 15 years [10] as in the current case. Of importance in the present case is that the migration was diagnosed 8 years prior to the admittance for acute presentation requiring surgical treatment. Same observation has been reported by Widmer et al. [13] with a case of known migration 10 years before the patient required surgery for small bowel obstruction. Distal dislocation of the band may therefore occur years after the erosion and migration within the stomach.

In the present case, initial clinical presentation oriented toward pancreatitis due to pain and blood amylase levels of more than 3 times the normal limit, a known biliary lithiasis and previous episode of pancreatitis both supported this hypothesis. Worsening of the clinical status under expectant management which necessitated a CT scan allowed the change of the initial diagnosis. An initial presentation as pancreatitis or biliary disorder has been reported in 2 case reports. In the case reported by Shah et al. [10], a migrated and dislocated band occluded the jejunum with associated duodenum wall thickening and signs of pancreatitis. Nasser et al. [14] has reported a case of biliary dilation with a narrowing of the lower bile duct likely secondary to compression from the tubing of a migrated band into the jejunum. The same mechanism on the papilla may be advocated in the present case in which however a rod like effect of the tube of the gastric band was extended to the pancreas as was revealed by the exploration of the lesser sac.

According to the literature data, life-threatening presentations of band erosion complications are very rare. As it occurred in the present case, 2 cases presenting with acute symptoms and peritonitis were reported in 2 case series [15], [16] in which the clinical picture was secondary to intestinal necrosis and associated gastric perforation [16]. In his article, Eun Young Kim [17] reported 2 cases of focal trans-mural gastric necrosis, several years following LAGB operation, necessitating total gastrectomy with Roux-En-Y reconstruction. In these cases, a strangulation mechanism of the stomach blood supply had been advocated. Other than peritonitis, reported life-threatening complications includes a case of acute haemorrhage reported by Torab et al. [24] requiring urgent treatment and endoscopy.

As reported in the present case, distal dislocation into the gastro-intestinal tract may complicate the erosion of the band within the stomach. Dislocation without major gastro-intestinal injuries has been reported in 3 case reports [10], [13], [14], all of which had the band removed trough an enterotomy, while only 2 reported performing a gastrostomy following the enterotomy [10], [14]. In the case reported by Widmer et al. [13], the jejunal segment was resected due to a conglomerate formation, no procedure was done on the stomach.

The jejunum is not the only distal dislocation reported in the literature. Corvini et al. [12] reported a case of migration of the band into the transverse colon and back to the stomach requiring a colon resection with primary anastomosis and a repair. Decubitus could be hypothesized in this case as in the case reported by Basam et al. [11] in which the band migrated further down to the rectum

Moreover, injuries secondary to the dislocation may be source of further complications requiring different surgical approaches. In 2 cases of injured gastro-intestinal tract, the reported lesion was local necrosis of the jejunum at the site where the band was dislocated [15], [16]. The maximum distance from the Treitz ligament was about 50−60 cm and both cases required a resection of the jejunum with primary anastomosis. According to the former observation (local necrosis observed at the site of migration of the band), and the distribution of lesions in the case we presented, it could be hypothesized that a combination of repeated decubitus in different sites aggravated by peristalsis may explain the observed skip lesions. These skip lesions were found starting from the stomach down to the small bowel at 90 cm from the Treitz ligament without involving the area of the papilla.

According to the study of Beitner et al. [19] intra-gastric migration of the band may be due to the configuration of the first-generation band providing high pressure in a limited narrow ring, distributed only on 325° of the circumference. New devices with lower pressure on a wider ring surface distributed on its entire 360°circumferences, are expected to dramatically reduce this complication, coupled with the rising experience of surgeons in the area. Moreover, due to disappointing long term results, LAGB is nowadays the less performed bariatric procedure [2]. However, considering the different reported timings of migration diagnosis (up to 18 years following surgery) [23] and the number of published series [9], [22], with large more recent series including more than 2000 patients [18], [19], [20], [21], [25], [26], additional similar cases could be expected in the future.

## 4 Conclusion

Although very rare, migration and distal dislocation of the band may occur as a late complication up to 18 years following LAGB, and according to the current data, additional similar cases may be expected in the future. Due to the clinical presentation, band migration and distal dislocation with gastro-intestinal injuries should always be considered in the differential diagnosis of abdominal complain in patients with gastric banding. Such complication could cause a severe clinical deterioration, an unexpected extension of gastro-intestinal injuries could furthermore require complex surgical approaches.

## SCARE

The work has been reported in line with the SCARE 2018 criteria [27].

## Declaration of Competing Interest

The authors disclose any financial and other personal relationships with other people or organisations that could inappropriately influence their work.

## Funding

No funding source. This is a self-financed manuscript.

## Ethical approval

As this is a case report, no ethical approval was necessary. Patient informed consent was given.

## Consent

Written informed consent was obtained from the patient for publication of this case report and accompanying images. A copy of the written consent is available for review by the Editor-in-Chief of this journal on request.

## Author contribution

Safi Khuri contributed to the writing of the manuscript as well as for the design. Subhi Mansour contributed as well for the design. Giuseppe Borzellino contributed to literature research and editing the manuscript. Yoram Kluger was the mentor and contributed to critical revision of the manuscript.

## Registration of research studies

N/A.

## Guarantor

Dr. Safi Khuri is the guarantor.

## Provenance and peer review

Not commissioned, externally peer-reviewed.
